# Multidisciplinary Management of Phenytoin-Induced Gingival Enlargement: A Case Report

**DOI:** 10.7759/cureus.101660

**Published:** 2026-01-16

**Authors:** Disha Gupta, Zoya Chowdhary, Shalabh Mehrotra, Bharti Chaudhary, Akash Bhatnagar

**Affiliations:** 1 Periodontology, Teerthanker Mahaveer Dental College and Research Centre, Moradabad, IND; 2 Dentistry, Government District Hospital, Reasi, IND; 3 Pediatric and Preventive Dentistry, Teerthanker Mahaveer Dental College and Research Centre, Moradabad, IND

**Keywords:** drug-induced oral manifestations, epilepsy, gingival enlargement, periodontal therapy, phenytoin

## Abstract

This case report describes a 21-year-old female patient with a history of epilepsy who developed generalized gingival enlargement after 18 months of phenytoin therapy. The patient presented with pain, difficulty in chewing, and esthetic concerns. Clinical examination revealed generalized edematous gingival overgrowth with bleeding on probing, pseudopockets, and subgingival calculus. A multidisciplinary approach was adopted, beginning with consultation with the patient’s physician regarding alternative medication. Non-surgical periodontal therapy, including scaling, root planing, and oral hygiene reinforcement, was performed to reduce inflammation. Following improvement in gingival condition, surgical management with an external bevel gingivectomy was carried out. Histopathological evaluation confirmed features consistent with phenytoin-induced gingival enlargement. Postoperative healing was satisfactory, and the patient was maintained on supportive periodontal therapy. This case highlights the importance of multidisciplinary collaboration between physicians and dental professionals in the management of drug-induced gingival enlargement. Comprehensive treatment that integrates pharmacologic modification with periodontal therapy can restore oral health, improve esthetics, and enhance patient confidence and quality of life.

## Introduction

Gingival enlargement is defined as an abnormal increase in the size of the gingival tissue, caused by systemic diseases, chronic inflammation, or drug therapy [1]. Among drug-induced gingival enlargement (DIGE), anticonvulsants such as phenytoin, calcium channel blockers such as nifedipine, and immunosuppressants such as cyclosporine are most frequently implicated [2]. Phenytoin, a commonly prescribed antiepileptic medication for seizure disorders, is reported to induce gingival enlargement in nearly 50% of patients receiving long-term therapy [3]. This condition negatively impacts oral hygiene, esthetics, and overall quality of life. Its estimated prevalence is 15-50% in the Indian population.

According to the 2017 World Workshop Classification, DIGE is categorized under non-plaque-induced gingival diseases, with phenytoin-induced gingival enlargement (PIGE) recognized as a specific subtype. Unlike non-DIGE, which arises from inflammatory, systemic, or neoplastic causes, DIGE is primarily medication-related but is modified by plaque-induced inflammation. This distinction is clinically relevant, as DIGE requires combined medical and periodontal management [4].

The pathogenesis of PIGE is multifactorial and involves drug-related changes in cellular metabolism, genetic susceptibility, and oral hygiene status. The hyperplastic response is linked to alterations in fibroblast function and other cellular elements of gingival tissues [5,6]. In addition, local factors such as plaque accumulation and the patient's inflammatory response contribute to disease severity and complicate management [7].

The management of PIGE requires a coordinated, multidisciplinary approach between dental professionals and the treating physician. Medical intervention may involve substituting alternative anticonvulsant medications with a lower risk of gingival hyperplasia [6]. Periodontal therapy includes both non-surgical and surgical options. Non-surgical therapy emphasizes plaque control, scaling, and root planing to minimize inflammation [6,8]. In advanced cases, surgical intervention such as gingivectomy is often necessary to restore function and esthetics [5]. Long-term success depends on patient compliance with home care education and motivation and supportive periodontal therapy to minimize recurrence and maintain gingival health.

In addition to conventional non-surgical and surgical management, recent evidence emphasizes the role of gingival phenotype in long-term stability, with a thin phenotype being more prone to inflammation and relapse. Adjunctive soft-tissue modulation techniques and biologic adjuncts have therefore been evaluated to improve gingival stability after surgical therapy. Biologic agents such as injectable platelet-rich fibrin and hyaluronic acid have shown potential in enhancing soft-tissue thickness, wound healing, and reducing recurrence risk [9,10].

## Case presentation

A 21-year-old female patient presented to the Department of Periodontics with a chief complaint of pain and difficulty in chewing for six months. The patient reported a medical history of epilepsy for three years and was on phenytoin (100 mg) and phenobarbitone (50 mg) twice daily for the past 18 months. She noticed gingival changes approximately six months after initiating phenytoin therapy.

**Figure 1:**
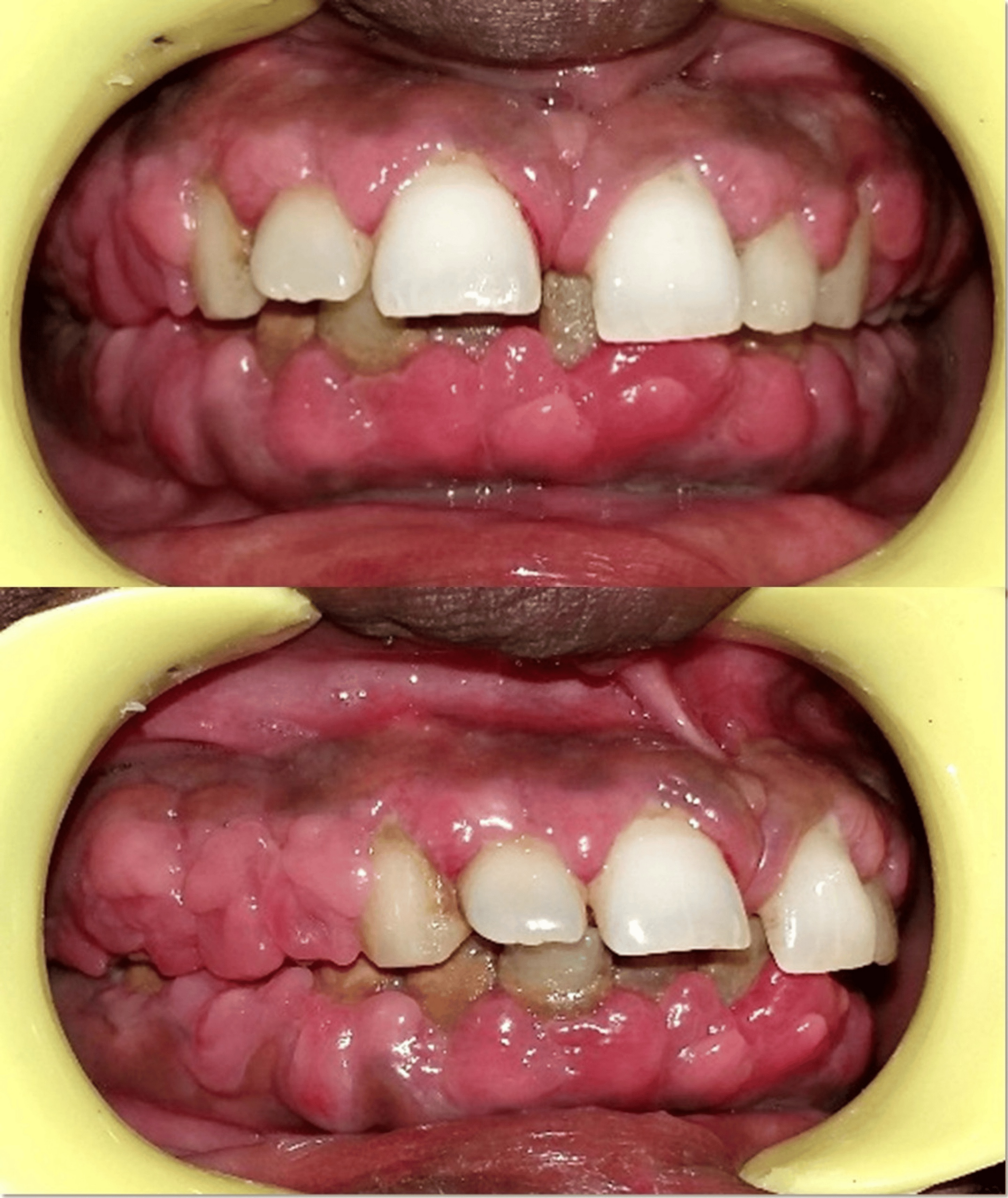
Preoperative view showing phenytoin-induced gingival enlargement

Clinical examination revealed generalized edematous gingival overgrowth involving both anterior and posterior regions. The enlargement extended to the occlusal surfaces of posterior teeth and from the incisal third to the middle third of anterior teeth (Figure 1). The gingiva appeared pinkish-red with marked bleeding on probing, suggestive of secondary inflammation. Generalized pseudopockets, tooth mobility, and subgingival calculus deposits were also observed.

**Figure 2:**
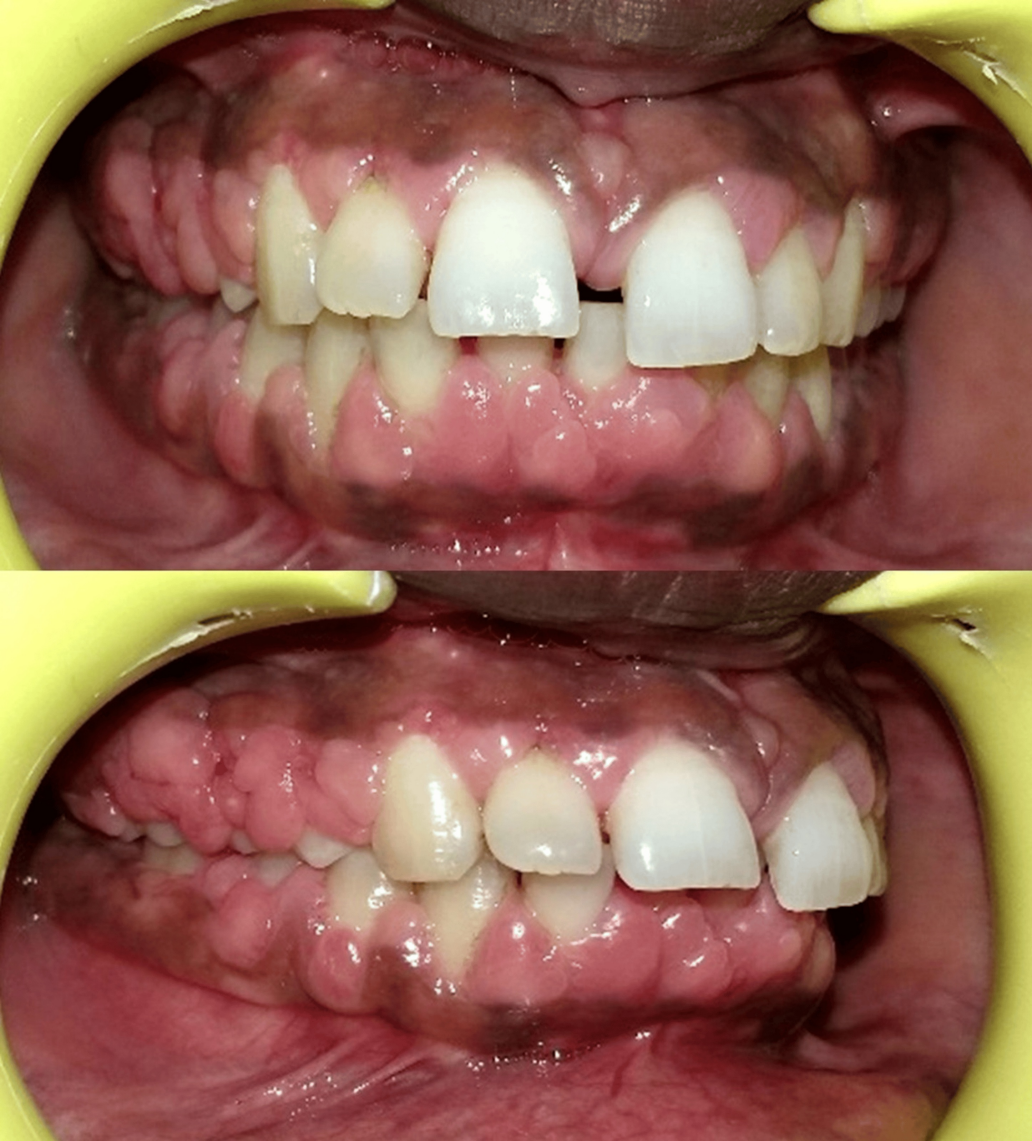
Post-scaling and root planing view demonstrating reduction in gingival inflammation

The patient was informed about the condition, and consultation with her physician was advised to explore possible alternatives to phenytoin. Blood investigations were performed to rule out systemic contraindications for periodontal surgery. Initial non-surgical periodontal therapy, including scaling, root planing, oral hygiene reinforcement, and prescription of 0.12% chlorhexidine mouthwash, was carried out. The patient was recalled after two months, during which a significant reduction in gingival inflammation and swelling was noted (Figure 2).

**Figure 3:**
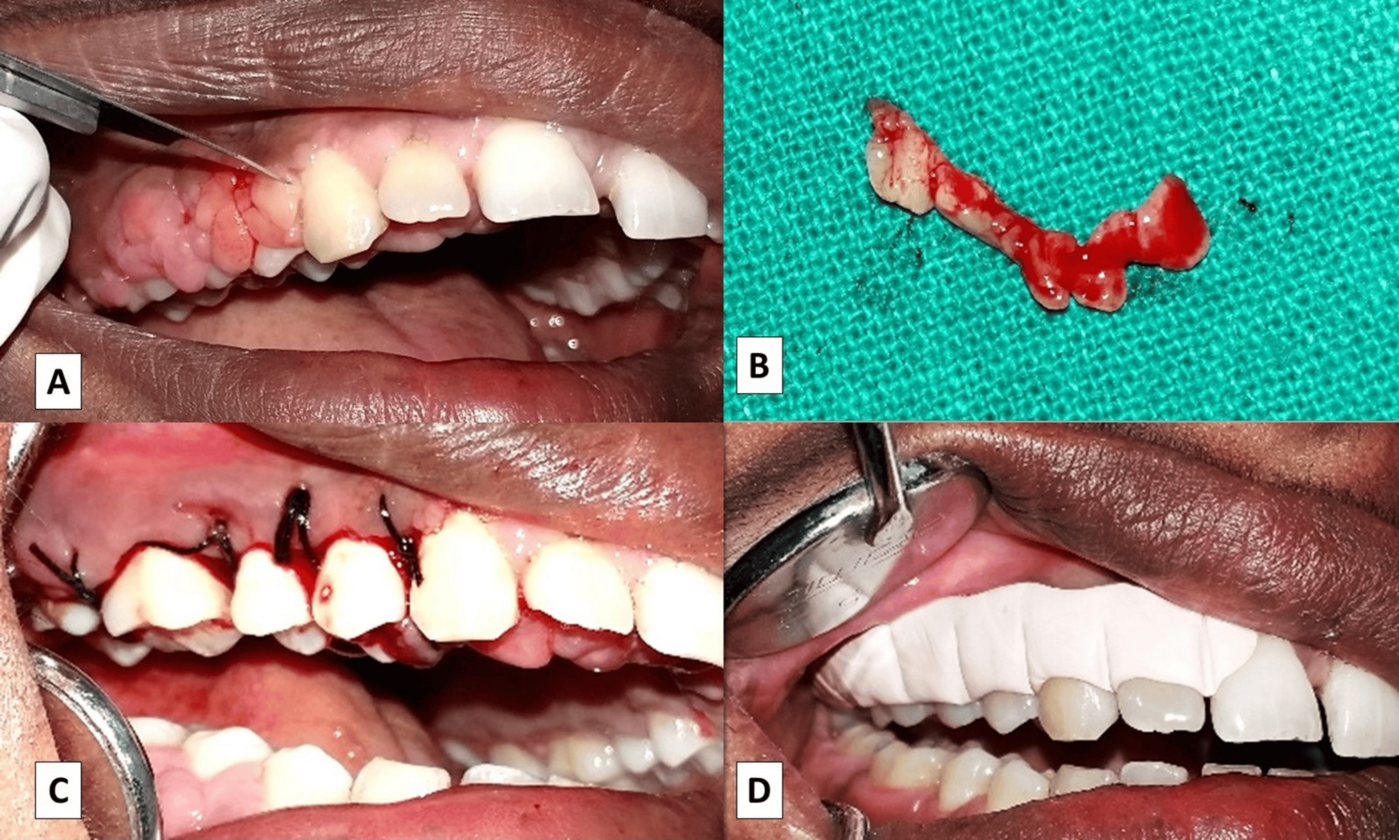
(A) An external bevel incision performed with a no. 11 blade. (B) An excised gingival tissue specimen. (C) Sutures placed following gingivectomy. (D) Periodontal dressing (COE-PAK™) applied at the surgical site

Surgical intervention was planned to address the persistent gingival enlargement. Under local anesthesia, bleeding points were marked using a Crane-Kaplan pocket marker. A continuous external bevel incision was placed with a no. 11 blade, maintaining the natural gingival contour (Figure 3A). Gingival tissue was excised, granulation tissue removed, and the surgical site thoroughly debrided. Excised tissue was fixed in 10% formalin and sent for histopathological evaluation (Figure 3B). Interrupted sutures were placed, followed by a periodontal dressing (COE-PAK™) (GC International AG, Lucerne, Switzerland) (Figure 3C, 3D).

**Figure 4:**
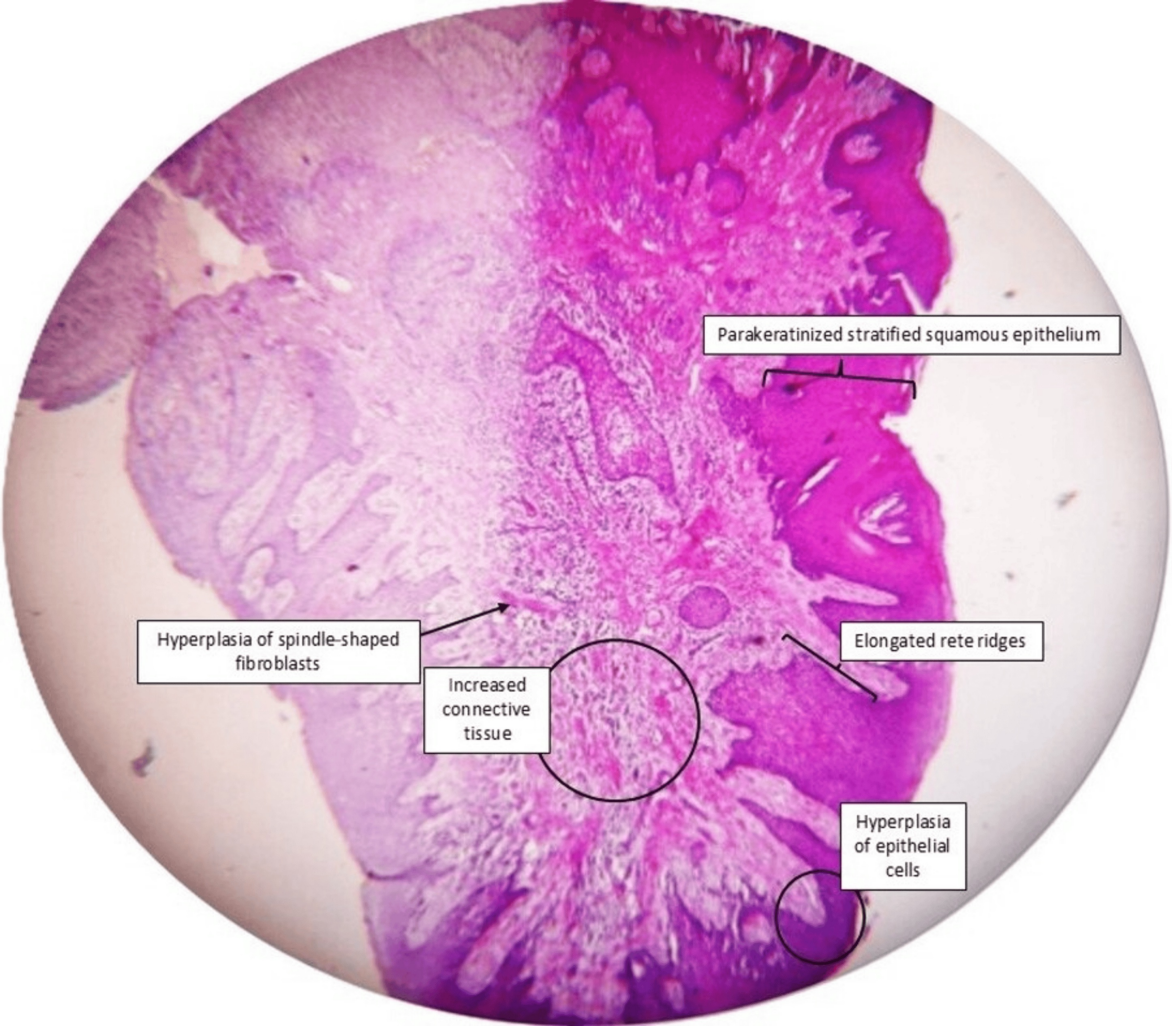
Histopathological image at 4× magnification showing parakeratinized stratified squamous epithelium with hyperplasia and elongated rete ridges

**Figure 5:**
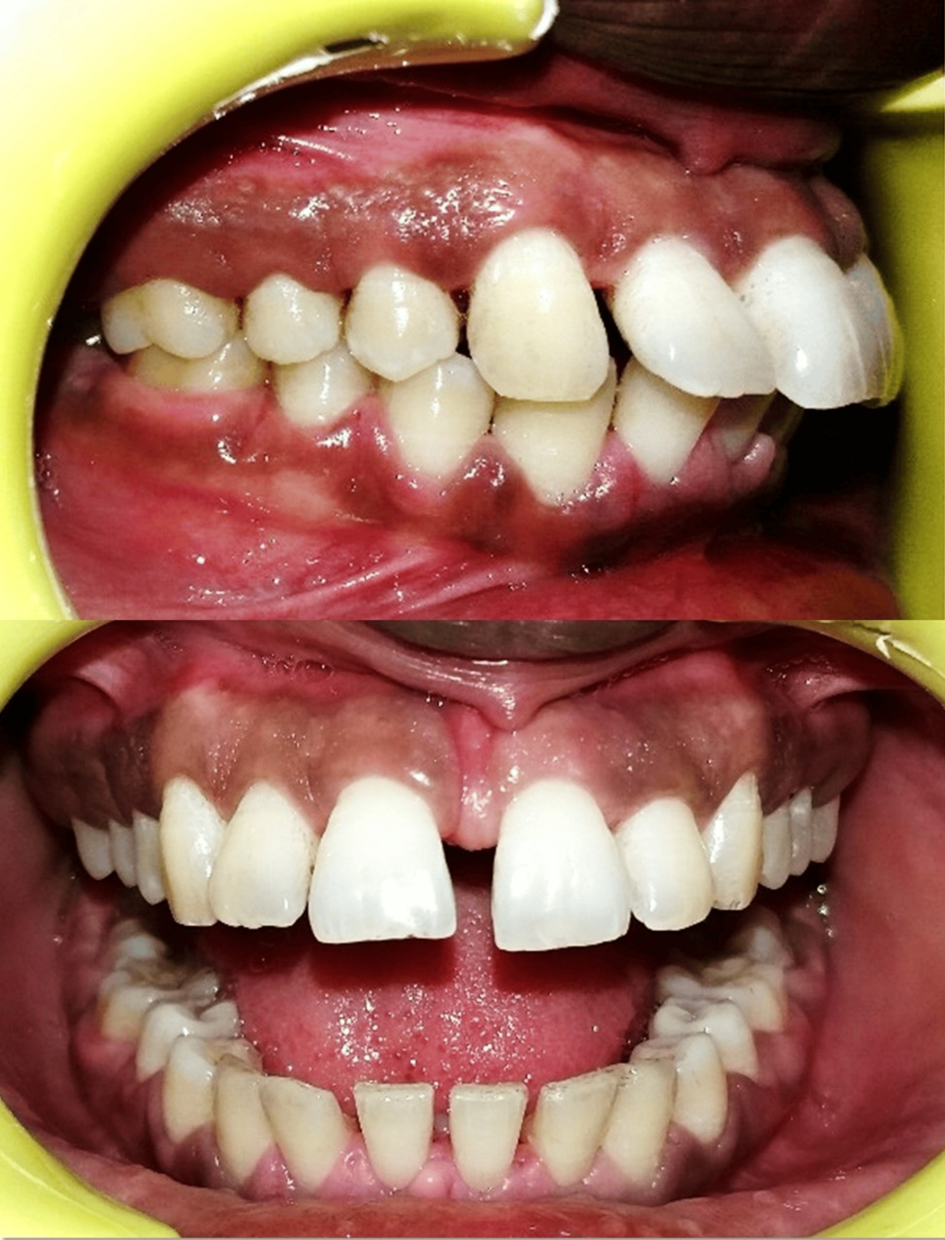
Six-month postoperative view demonstrating satisfactory healing and stable gingival architecture

Postoperative care included antibiotics, analgesics, and oral hygiene instructions. The patient demonstrated satisfactory healing and was placed on supportive periodontal therapy. Histopathology revealed parakeratinized stratified squamous epithelium with hyperplasia and elongated rete ridges projecting into the connective tissue. The underlying stroma showed increased collagen fibers, spindle-shaped fibroblasts, and a dense inflammatory infiltrate predominantly composed of lymphocytes and plasma cells, with multiple blood vessels (Figure 4). At six months postoperatively, satisfactory healing and stability of gingival architecture were observed (Figure 5).

## Discussion

Phenytoin remains one of the most commonly prescribed anticonvulsant medications for epilepsy, although its long-term use is strongly associated with gingival enlargement [2]. Other drugs linked to this condition include calcium channel blockers and immunosuppressants [2]. The prevalence of PIGE has been reported in nearly half of long-term users [3]. In addition to functional impairment, the esthetic concerns associated with gingival enlargement often have significant psychosocial implications for patients.

The pathogenesis of PIGE is multifactorial. Altered fibroblast activity and excessive extracellular matrix deposition are considered central mechanisms [11,12]. Several signaling pathways, such as transforming growth factor-beta (TGF-β), have been implicated in stimulating fibroblast proliferation and collagen synthesis while reducing collagen degradation [13]. Genetic variations, particularly in drug-metabolizing enzymes such as CYP2C9, may also influence susceptibility [14]. Furthermore, inflammatory mediators including interleukin-6 (IL-6) and tumor necrosis factor alpha (TNF-α), as well as local factors such as plaque accumulation, exacerbate gingival overgrowth [13,15].

The present case highlights the importance of a multidisciplinary approach in the management of PIGE. Collaboration with the patient's physician was essential to evaluate potential modifications in anticonvulsant therapy while maintaining seizure control. Initial non-surgical therapy was performed to minimize plaque-induced inflammation, followed by a gingivectomy to address persistent enlargement. This sequence of care supports existing evidence that surgical intervention is often required in moderate-to-severe cases when non-surgical measures alone are insufficient [6,8]. Postoperative maintenance is crucial in preventing recurrence and ensuring long-term stability. Daily plaque control through proper toothbrushing, interdental cleaning, and use of adjunctive antimicrobial mouth rinses helps minimize gingival inflammation, which is a major modifying factor in the progression and recurrence of gingival overgrowth. When combined with regular supportive periodontal therapy, good home care practices help preserve the surgically achieved gingival contour, enhance periodontal stability, and significantly reduce the risk of recurrence.

Alternative pharmacologic options, such as carbamazepine and valproic acid, are associated with a lower risk of gingival overgrowth and may be considered in selected patients [16]. Additionally, folic acid supplementation has been shown to reduce the severity of phenytoin-induced gingival changes, particularly in children [17]. Nevertheless, effective plaque control remains a cornerstone of management, as inadequate oral hygiene significantly increases the severity of enlargement.

Recurrence is influenced by continued exposure to the offending drug, inadequate plaque control, and irregular supportive periodontal therapy. Even after successful surgical intervention, persistent inflammatory stimuli from poor oral hygiene can reactivate fibroblastic proliferation and extracellular matrix accumulation, leading to the regrowth of gingival tissue. Substitution of phenytoin with alternative anticonvulsant medication may significantly reduce, but not completely eliminate, the risk of recurrence. Therefore, long-term maintenance through strict home care practices, regular periodontal follow-up, and close medical-dental collaboration is essential to monitor gingival health, detect early signs of relapse, and ensure sustained clinical stability [18].

This case underscores the role of dental professionals in the early recognition and management of DIGE. Comprehensive care, involving both medical and periodontal interventions, can restore oral function and esthetics while improving overall quality of life. A multidisciplinary approach ensures optimal patient outcomes by addressing both the pharmacologic and periodontal components of the condition.

Meticulous home care and compliance with supportive periodontal therapy are essential for maintaining treatment outcomes in PIGE. Effective plaque control reduces gingival inflammation and the recurrence of disease. Regular maintenance visits facilitate the early detection of recurrent changes and reinforcement of oral hygiene practices. Despite successful surgery, recurrence remains a risk, particularly with poor oral hygiene or continued drug exposure, emphasizing the need for frequent follow-up [18,19].

## Conclusions

PIGE is a well-recognized complication of long-term anticonvulsant therapy that can significantly affect oral function, esthetics, and quality of life. Effective management requires both pharmacologic and periodontal interventions, highlighting the importance of collaboration between physicians and dental professionals. This case demonstrates that a multidisciplinary approach combining non-surgical therapy, surgical intervention, and supportive periodontal care can achieve stable long-term outcomes. Early recognition, patient education, and regular maintenance are critical to preventing recurrence and improving overall treatment success.
